# Results of salvage therapy with mini-hyper-CVD and inotuzumab ozogamicin with or without blinatumomab in pre-B acute lymphoblastic leukemia

**DOI:** 10.1186/s13045-023-01444-2

**Published:** 2023-05-02

**Authors:** Hagop Kantarjian, Fadi G. Haddad, Nitin Jain, Koji Sasaki, Nicholas J. Short, Sanam Loghavi, Rashmi Kanagal-Shamanna, Jeffrey Jorgensen, Issa Khouri, Partow Kebriaei, Yesid Alvarado, Tapan Kadia, Shilpa Paul, Guillermo Garcia-Manero, Bouthaina Dabaja, Musa Yilmaz, Jovitta Jacob, Rebecca Garris, Susan O’Brien, Farhad Ravandi, Elias Jabbour

**Affiliations:** 1 Department of Leukemia, The University of Texas MD Anderson Cancer Center, 1515 Holcombe Blvd., Box 428, Houston, TX 77030 USA; 2 Department of Hematopathology, The University of Texas MD Anderson Cancer Center, Houston, TX 77030 USA; 3 Department of Stem Cell Transplantation, The University of Texas MD Anderson Cancer Center, Houston, TX 77030 USA; 4 Department of Pharmacy, The University of Texas MD Anderson Cancer Center, Houston, TX 77030 USA; 5 Department of Radiation Oncology, The University of Texas MD Anderson Cancer Center, Houston, TX 77030 USA; 6 Chao Family Comprehensive Cancer Center, University of California Irvine, Orange, CA USA

**Keywords:** Philadelphia-negative ALL, Inotuzumab, Blinatumomab, Chemo-immunotherapy, Salvage, Outcome

## Abstract

**Background:**

Historically, adults with relapsed-refractory acute lymphoblastic leukemia (ALL) experienced poor outcomes with intensive chemotherapy. This mature analysis explores the benefit of the addition of sequential blinatumomab to low-intensity mini-Hyper-CVD chemotherapy with inotuzumab ozogamicin in this setting.

**Methods:**

Mini-Hyper-CVD (cyclophosphamide and dexamethasone at 50% dose reduction, no anthracycline, methotrexate at 75% dose reduction, cytarabine at 83% dose reduction) was combined with inotuzumab during the first 4 courses. From Patient #68 and onwards, inotuzumab was given in reduced and fractionated doses, and blinatumomab was added sequentially for 4 courses. Maintenance therapy with prednisone, vincristine, 6-mercaptopurine and methotrexate was given for 12 courses, and blinatumomab for 4 additional courses.

**Results:**

Among 110 patients (median age, 37 years) treated, 91 (83%) responded (complete response, 69 patients, 63%). Measurable residual disease negativity was documented in 75 patients (82% of responders). Fifty-three patients (48%) received allogeneic stem cell transplantation (SCT). Hepatic sinusoidal obstruction syndrome occurred in 9/67 patients (13%) on the original inotuzumab schedule and in 1/43 (2%) on the modified schedule. With a median follow-up of 48 months, the median overall survival (OS) was 17 months, and the 3 year OS was 40%. The 3 year OS was 34% with mini-Hyper-CVD plus inotuzumab and 52% with additional blinatumomab (*P* = 0.16). By landmark analysis at 4 months, the 3 year OS was 54%, similar between patients who did or did not receive allogeneic SCT.

**Conclusion:**

Low-intensity mini-Hyper-CVD plus inotuzumab with or without blinatumomab showed efficacy in patients with relapsed-refractory ALL, with better survival after the addition of blinatumomab.

*Trial registration* The trial was registered on clinicaltrials.gov with the identifier NCT01371630.

**Supplementary Information:**

The online version contains supplementary material available at 10.1186/s13045-023-01444-2.

## Introduction

The introduction of targeted therapies like the BCR::ABL1 tyrosine kinase inhibitors in Philadelphia-chromosome(Ph)-positive acute lymphoblastic leukemia (ALL), of immune-oncologic therapies like antibodies targeting CD19, CD20 and CD22, as well as chimeric antigen receptor T (CAR-T) cellular therapies in pre-B ALL, is transforming the therapeutic landscape in adult ALL [1–3]. This research has already resulted in the Food and Drug Administration (FDA) approvals of blinatumomab (CD19 bi-specific T-cell engager [BiTE]; approved in 2014), inotuzumab ozogamicin (antibody–drug conjugate targeting CD22; approved in 2017), and two CARTs (tisagenlecleucel approved in 2017; brexucabtagene autoleucel approved in 2021) as ALL salvage therapies [4–7].

Single-arm and later randomized trials confirmed the efficacy of inotuzumab and blinatumomab as single-agent therapies in relapsed-refractory (R-R) ALL [4, 5]. In this setting, inotuzumab therapy resulted in an overall response rate of 80% and a median overall survival (OS) of 7.7 months [5]. Blinatumomab resulted in an overall response rate of 44% and a median OS of 7.7 months [4]. Better results were achieved when the treatments were given in earlier salvage conditions and in ALL in remission but with measurable residual disease (MRD) [8–10].

To improve the results, we combined low intensity chemotherapy (mini-Hyper-CVD) with inotuzumab. We have previously reported that this regimen resulted in an overall response rate of 80% and a median OS of 11 months. A post hoc analysis from that study showed improved survival with the combination compared with single-agent inotuzumab (median OS, 9.3 vs. 5.6 months; *P* = 0.02) [11]. We then reduced and fractionated the inotuzumab doses and added sequential blinatumomab for 4 courses and 3 additional later courses during maintenance therapy [12]. The aim of this approach was to reduce intensive chemotherapy, make inotuzumab safer while maintaining its efficacy, improve the depth of MRD responses, and distance allogeneic stem cell transplantation (SCT) from the last dose of inotuzumab, in order to reduce treatment-related morbidity, hepatic sinusoidal obstruction syndrome (SOS) and mortality. In this updated analysis with a median follow-up of 48 months, we report the long‐term results of this regimen (mini-Hyper-CVD-inotuzumab ± blinatumomab) in 110 patients, and assess the impact of reducing and fractionating inotuzumab, and of adding sequential blinatumomab, on the long-term outcome of patients with R-R ALL.

## Methods

### Study design and participants

Patients with R-R Philadelphia chromosome-negative CD22-positive pre-B ALL were eligible. Patients had to have an Eastern Cooperative Oncology Group (ECOG) performance status of 3 or better, normal cardiac function (defined by an ejection fraction above 50%), and adequate organ functions (serum bilirubin ≤ 1.95 mg/dL and serum creatinine ≤ 2.0 mg/dL). Patients were excluded if they had an active infection not controlled by antibiotics, clinical evidence of grade 3 to 4 heart failure as defined by the New York Heart Association criteria, or second active malignancy. All patients signed a consent form in accordance with the Declaration of Helsinki. This study was approved by the Institutional Review Board of The University of Texas MD Anderson Cancer Center. The trial was registered on clinicaltrials.gov with the identifier NCT01371630.

### Procedures

The details of the regimen have been previously published [11–17]. The induction and odd courses (Courses 1, 3, 5, 7) included cyclophosphamide (150 mg/m^2^ every 12 h on Days 1–3) and dexamethasone (20 mg per day on Days 1–4 and 11–14) given at 50% dose reduction; no anthracycline was administered. Vincristine (2 mg flat dose) was given on Day 1 and 8. The even courses (Courses 2, 4, 6, 8) delivered methotrexate 250 mg/m^2^ on Day 1 (75% dose reduction) and cytarabine 0.5 g/m^2^ given every 12 h on Days 2 and 3 (83% dose reduction). Prior to an amendment designed to reduce the inotuzumab toxicity (Patient #1 to Patient #67), inotuzumab was administered on Day 3 of each of the first 4 courses. Inotuzumab was given at 1.8–1.3 mg/m^2^ in Course 1 followed by 1.3–1.0 mg/m^2^ during the subsequent 3 courses. The cumulative total inotuzumab planned dose was 4.3–5.7 mg/m^2^. Courses were administered every 4 weeks for a total of 8 (Additional file 1: Fig. S1A).

Rituximab was given on Days 1 and 11 of Courses 1 and 3, and on Days 1 and 8 of Courses 2 and 4 (total 8 doses) in patients with CD20 expression ≥ 20%. Central nervous system (CNS) prophylaxis consisted of intrathecal therapy with methotrexate and cytarabine given alternately on Days 2 and 7 (± 3 days) of each course for a total of 8 doses. The order of intrathecal chemotherapy was reversed with the even courses: cytarabine on Day 2 and methotrexate on Day 7 (to avoid simultaneous systemic and intrathecal methotrexate, which might rarely cause demyelination and neurotoxicity) [18, 19]. For patients presenting with active CNS disease, confirmed by cytologic examination of the cerebrospinal fluid (CSF), triple intrathecal therapy (TIT; cytarabine 40 mg, methotrexate 6 mg via Ommaya reservoir, 12 mg IT; hydrocortisone 50 mg) was repeated twice weekly until the CSF became clear of leukemic cells. Patients then received TIT once a week for 4 weeks or until initiation of the next course of chemotherapy, when the regimen was resumed.

Maintenance therapy was given for 3 years with monthly vincristine 2 mg for 1 year, prednisone 50 mg daily for 5 days every month for 1 year, 6-mercaptopurine 50 mg twice daily for 3 years, and methotrexate 10 mg/m^2^ orally weekly for 3 years (POMP regimen). Initiation of maintenance due to treatment-related toxicity prior to completion of the consolidation phase (Courses 2–8) was allowed. Dose reductions of the cytotoxic agents according to the type and degree of side effects or toxicity were permitted and followed previously published guidelines [16]. Proceeding with allogeneic SCT was at the discretion of the treating physician after discussion with the patient. Factors considered were usually the salvage status, the achievement of a negative MRD status, the risk of SOS, and whether SCT can be performed in remission.

To reduce the risk of SOS and improve outcome, the protocol was amended in February 2017 to use fractionated and lower doses of inotuzumab, reduce chemotherapy from 8 to 4 courses, and add sequentially 4 courses of blinatumomab, followed by blinatumomab every 3 months × 4 during POMP maintenance (Additional file 1: Fig. S1B). The amendments started with Patient # 68. This was based on studies showing the fractionated weekly lower dose schedule of inotuzumab to be safer and as effective as the single monthly schedule [18, 19]. After this amendment, inotuzumab was given as 0.6 mg/m^2^ on Day 2 and 0.3 mg/m^2^ on Day 8 of Course 1, and as 0.3 mg/m^2^ on Day 2 and 0.3 mg/m^2^ on Day 8 in Courses 2, 3, 4. The total cumulative planned dose was 2.7 mg/m^2^. The 4 courses of mini-Hyper-CVD plus inotuzumab were followed by 4 courses of blinatumomab (Courses 5–8). Maintenance therapy was reduced to 12 courses of POMP with one course of blinatumomab after every 3 courses of POMP for a total of 4 courses. Blinatumomab was given by continuous infusion at 9 mcg/day in the first 4 days of Course 1 then escalated to 28 mcg/day by Day 5 for the rest of the 28 days in Course 1. It was then given at 28 mcg/day for 4 weeks in the subsequent courses. Courses were 6 weeks (4 weeks on, 2 weeks off) [12, 15, 17].

Supportive care measures were according to the institutional standard guidelines. Monitoring for tumor lysis and prophylaxis with allopurinol, or alternatives such as rasburicase, and appropriate intravenous hydration were administered in the first course to all patients. All patients received prophylactic antimicrobial therapy (levofloxacin or cefpodoxime; azole; valacyclovir or acyclovir) during neutropenia, which began in induction. Azoles, usually voriconazole or posaconazole, were held on Day-1, Day 0, and Day+1 of the vincristine administration to avoid increased vincristine neurotoxicity. Pegfilgrastim 6 mg subcutaneously was administered on Day 4 (+ 2 days) of each of the induction/consolidation courses. Ursodiol 300 mg orally 3 × daily was given as SOS prophylaxis since the protocol was amended in September 2015.

### Outcomes

The primary endpoints of the analysis were overall response rate (including complete remission [CR], CR with incomplete platelet recovery [CRp], and CR with incomplete hematologic recovery [CRi]) and OS. Landmark analysis for OS was reported at the 2 months and 4 months cutoffs for patients receiving blinatumomab (the median time to receiving blinatumomab was 2 months) and allogeneic SCT (the median time to allogeneic SCT was 4 months), respectively. Secondary endpoints included safety measures, relapse-free survival (RFS), the rate of subsequent allogeneic SCT, and the MRD negativity rate. Response to therapy was by bone marrow evaluations after Course 1 then after every 2–4 courses of consolidation and every 3–6 months during maintenance. CR was defined as the presence of ≤ 5% blasts in the bone marrow, with more than 1 × 10^9^/L neutrophils, more than 100 × 10^9^/L platelets in the peripheral blood, and no extramedullary disease. CRp was defined as CR except for platelets less than 100 × 10^9^/L. CRi was defined as CR but with an absolute neutrophil count of less than 1 × 10^9^/L neutrophils and platelets less than 100 × 10^9^/L. MRD assessment using clinically validated multicolor/multiparameter flow cytometry was performed on whole bone marrow specimens [20, 21]. MRD negativity was defined as undetectable leukemic blasts by multicolor/multiparameter flow cytometry at a sensitivity of 1 × 10^–4^.

We performed in situ hybridization (FISH) technique using a CRLF2 dual color, breakapart DNA probe from Cytocell Ltd. The probe hybridized to band Xp22.33/Yp11.32 to detect CRLF2 rearrangement. We performed a next-generation sequencing-based analysis for the detection of somatic mutations in the coding sequence of the *TP53* gene on the DNA extracted from samples. MRD was also assessed by next-generation sequencing (NGS) with a sensitivity of 1 × 10^–6^ (ClonoSEQ MRD assay; Adaptive Biotechnologies Co., Seattle, WA) in patients with available bone marrow samples.

Relapse was defined as recurrence of more than 5% lymphoblasts in a bone marrow aspirate unrelated to recovery, or by the presence of extramedullary disease. RFS was calculated from the time of CR until relapse or death. OS was calculated from the time of treatment initiation until death.

Adverse events were defined as any event that occurred between the first dose and 2 months after the last dose, all treatment-related events that occurred after the last dose, and all cases of SOS (of any cause) that occurred within 2 years after inotuzumab therapy. SOS was assessed, diagnosed and evaluated according to previously defined clinical criteria [22].

### Statistical analysis

This is a phase II study in R-R pre-B ALL in which 110 consecutive patients were treated. The trial was continuously monitored, with an early stopping rule in place if it was ever likely that the trial’s OS was less than that of previous similar trials. No stopping rules were met. Survival curves were plotted by the Kaplan–Meier method and compared with the log-rank test. Differences in subgroups were evaluated with the Chi-squared test for nominal values and the Mann–Whitney U and Fisher exact tests for continuous variables. A *P*-value of < 0.05 (two-tailed) was considered statistically significant. We performed univariate and multivariate Cox regression analysis to identify prognostic factors for survival, with a *P*-value cutoff of 0.05 from univariate to multivariate analysis.

### Role of the funding source

The funder provided free study drug and funding for a research nurse for this study. The funder had no role in the study design, monitoring, data collection, data analysis and interpretation, or writing of the study. H.K and E.J. had full access to all the data in the study and final responsibility for the publication.

## Results

From November 12, 2012 to July 23, 2021, 110 patients were treated (Additional file 1: Fig. S2). Their characteristics are shown in Table 1. Their median age was 37 years (range, 17–87 years). Overall, 108/110 patients (98%) had received prior frontline intensive chemotherapy, including a Hyper-CVAD regimen or its variant in 76 patients (69%) and others in 32 patients (29% — CALGB 8%, Augmented BFM 6%, COG 4%, others). Sixty-seven patients (61%) were registered on mini-Hyper-CVD plus inotuzumab, and 43 patients (39%) on mini-Hyper-CVD with fractionated inotuzumab on Days 2 and 8 followed by blinatumomab. Four of 67 patients (6%) treated with mini-Hyper-CVD plus inotuzumab were subsequently taken off study and received blinatumomab. In the post amendment cohort, 32 of 43 patients (74%) received blinatumomab (two of them after being taken off study). Seventy-nine patients (72%) were in Salvage 1 and 38 (35%) had a first CR duration of more than 12 months. Thirty-one patients (28%) were in Salvage 2+. Twenty-one patients (19%) had failed prior allogeneic SCT. Twenty-two patients (20%) had high-risk cytogenetics including low-hypodiploidy/near triploidy in 12 patients (11%) and *KMT2A* rearrangements in 10 (9%); thirteen patients (12%) had complex karyotype. Twelve of the 71 patients (17%) tested had CRLF2 overexpression by FISH or flow cytometry. Nineteen of the 60 patients (32%) tested had *TP53* mutations. Six patients (5%) had CNS disease at the start of treatment. The median fraction of leukemic blasts with CD22 expression was 95.4% (range, 14.3–100%) and with CD19 expression was 99.9% (range, 0.5–100%). Twenty-eight patients (25%) had CD20 expression ≥ 20% and received rituximab during the first 4 courses.

**Table 1:** Patient characteristics (N = 110)

Characteristic	Category	N (%)/median [range]			*P*
		Overall (n = 110)	Before amendment* (n = 67)	After amendment* (n = 43)	
Age (years)		37 (17–87)	34 (17–87)	42 (18–79)	0.02
Gender	Male	52 (47)	31 (46)	21 (49)	0.80
ECOG performance status	≥ 2	19 (17)	11 (16)	8 (19)	0.76
WBC (× 10^9^/L)	Median	3.4 (0.1–194.7)	3.7 (0.1–194.7)	3.1 (0.8–129.9)	0.78
	≥ 50	4 (4)	2 (3)	2 (5)	0.65
PB blasts percentage		2.5 (0–97)	3 (0–93)	2 (0–97)	0.94
BM blasts percentage		70 (6–98)	72 (8–98)	50 (6–96)	0.28
BM blasts ≥ 50%		69 (63)	47 (70)	22 (51)	0.04
Karyotype	Diploid	28 (25)	14 (21)	14 (33)	0.17
	Other	26 (24)	17 (25)	9 (21)	0.60
	Complex	13 (12)	10 (15)	3 (7)	0.21
	*KMT2A* rearrangement	10 (9)	8 (12)	2 (5)	0.19
	Ho-tr	12 (11)	4 (6)	8 (19)	0.04
	HeH	3 (3)	3 (4)	0	0.16
	Tt	2 (2)	1 (1)	1 (2)	0.75
	IM/ND	16 (15)	10 (15)	6 (14)	0.89
High-risk cytogenetics**		22 (20)	12 (18)	10 (23)	
Ph-like (RNA sequencing)		20 (18)	7 (10)	13 (30)	
*CRLF2* overexpression***		12/71 (17)	6/34 (18)	6/37 (16)	0.87
*TP53* mutation		19/60 (32)	9/24 (38)	10/36 (28)	0.61
CD22 expression	Median	95.4 (14.3–100)	95.6 (20–100)	95.2 (14.3–99.9)	0.90
CD19 expression	Median	99.9 (0.5–100)	99.9 (0.5–100)	99.9 (10.5–100)	0.74
CD20 expression	≥ 20%	28 (25)	12 (18)	16 (37)	0.02
Prior ASCT		21 (19)	19 (28)	2 (5)	0.002
Salvage status	Salvage 1	79 (72)	38 (57)	41 (95)	< 0.001
	Salvage 1, primary refractory	15 (14)	5 (7)	10 (23)	
	Salvage 1, CRD1 < 12 months	26 (24)	17 (25)	9 (21)	
	Salvage 1, CRD1 ≥ 12 months	38 (35)	16 (24)	22 (51)	
	Salvage 2	17 (15)	15 (22)	2 (5)	
	≥ Salvage 3	14 (13)	14 (21)	0	

*Before the amendment, mini-Hyper-CVD + inotuzumab ozogamicin; after the amendment, mini-Hyper-CVD + inotuzumab ozogamicin + blinatumomab

**High-risk cytogenetics include low hypodiploidy/near triploidy and *KMT2A* rearrangement

***By FISH or flow cytometry

*ASCT* Allogeneic stem cell transplantation; *BM* Bone marrow; *CRD* Complete remission duration; *CRLF2* Colony receptor like factor 2; *ECOG* Eastern cooperative oncology group; *HeH* High hyperdiploidy; *Ho-Tr* Low hypodiploidy/near triploidy; *IM* Insufficient metaphases; *ND* Not determined; *PB* Peripheral blood; *Ph* Philadelphia-chromosome; *Tt* Tetraploidy; *WBC* White blood cell

Overall, patients received a median of 2 courses of induction/consolidation therapy (range, 1–8). Thirty-six patients (33%) received a median of 2 courses of blinatumomab (range, 1–4 courses). Twenty-one patients (19%) received all four planned courses of inotuzumab, and 5/43 patients (12%) received all 4 planned courses of blinatumomab.

Overall, 91 of the 110 patients (83%) responded, with a median time to response of 27 days (range, 12–134): CR in 69 (63%), CRp in 19 (17%), CRi in 3 (3%) (Table 2). Seventy-three of 91 patients (80%) responded after the first cycle of therapy, and 18 (20%) after subsequent cycles.

**Table 2:** Best overall response

Parameter	N (%)			*P*
	Overall (n = 110)	Before amendment (n = 67)	After amendment (n = 43)	
Morphologic response				
CR	69 (63)	40 (60)	29 (67)	0.41
CRp	19 (17)	10 (15)	9 (21)	0.42
CRi	3 (3)	1 (1)	2 (5)	0.32
ORR	91 (83)	51 (76)	40 (93)	0.02
MRD negativity				
At response	47/87 (54)	28/49 (57)	19/38 (50)	0.51
Overall	75/89 (84)	41/50 (82)	34/39 (87)	0.51
No response	12 (11)	9 (13)	3 (7)	0.29
Early death	7 (6)	7 (10)	0	0.03
MRD negativity				
Salvage 1				
At response	41/70 (59)	23/33 (70)	18/37 (49)	0.08
Overall	65/73 (89)	31/34 (91)	34/39 (87)	0.59
≥ Salvage 2				
At response	6/17 (35)	5/16 (31)	1/1 (100)	0.17
Overall	12/18 (67)	10/16 (63)	2/2 (100)	0.29

*CR* Complete response; *CRi* CR with incomplete hematologic recovery; *CRp* CR without platelets recovery; *MRD* Measurable residual disease; *ORR* Overall response rate

Seven patients (6%) died within 4 weeks of the start of therapy. The median time to death was 15 days (range, 4–26 days). Six of the 7 patients were in Salvage 2+; 2 had a performace status of 2. All 7 early deaths occurred before the protocol amendment (which could reflect the effect of the single higher dose inotuzumab or patient selection after the amendment).

Fifty-three patients (48%) underwent allogeneic SCT after a median of 4 months (range, 1.8–10.2 months). Among 91 patients who responded, 87 were assessed for MRD status at the time of morphologic response. The MRD negativity rate at the time of morphologic response was 54%. The best MRD negativity rate at any time within 3 courses was 84%. Overall, 36 complete cytogenetic responses were noted among the 39 patients (92%) with morphologic response and abnormal pretreatment karyotype. Response by Salvage status and duration of prior CR are shown in Additional file 1: Table S1. The overall response rate was 92% for patients treated in Salvage 1 (95% in the 36 patients with CR1 duration > 12 months). The overall response rates for patients treated in Salvage 2 and Salvage 3+ were 59% and 57%, respectively. Higher MRD negative response rates were obtained in patients treated in Salvage1 (89%) compared with Salvage 2 + (67%).

**Fig. 1:**
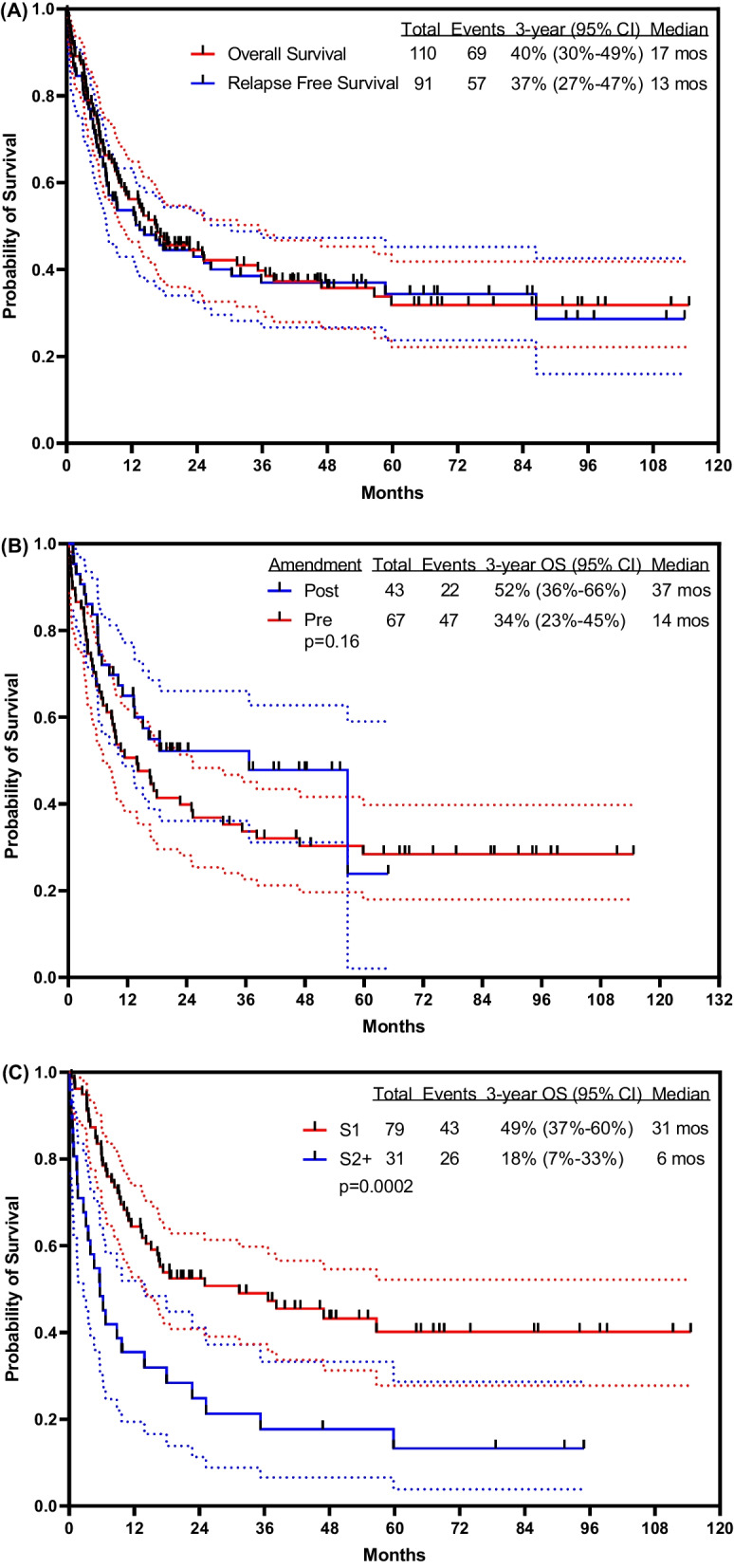

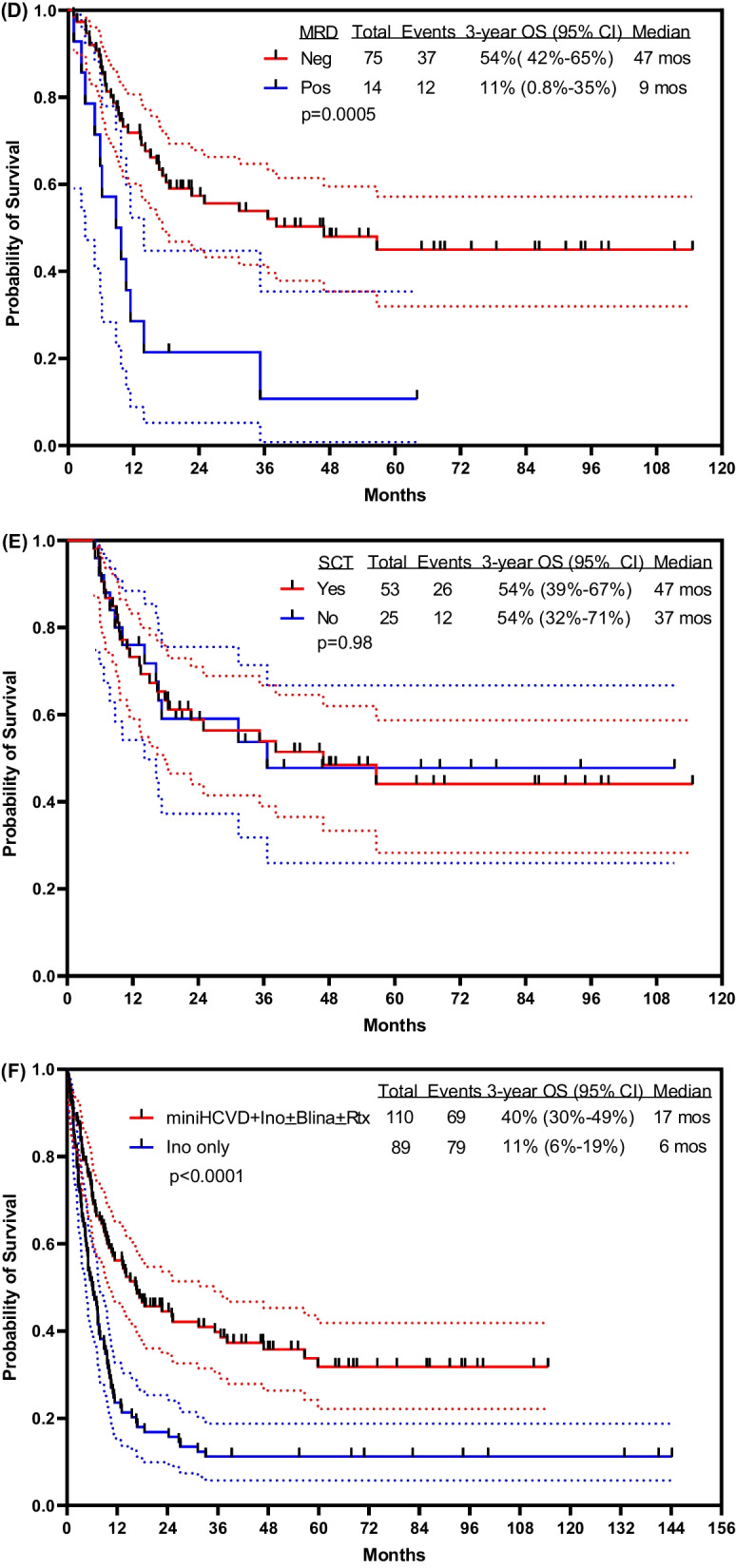
Survival outcome **A** overall, **B** by treatment modality, **C** by Salvage status, **D** by best MRD status, **E** by allogeneic stem cell transplantation, and **F** in comparison with inotuzumab monotherapy. *S1* Salvage 1; *S2* + Slavage 2 and beyond; *Neg* Negative; *Pos* Positive; *Ino* Inotuzumab; *Blina* Blinatimomab; *Rtx* Rituximab

With a median follow-up of 48 months (range, 9–115 months), 41 patients (37%) were alive, 34 (31%) in CR (24 post allogeneic SCT). The estimated 3 year OS rate was 40% [95% confidence interval (CI) 30–49%]. The estimated 3 year RFS rate was 37% (95% CI 27–47%). The median OS was 17 months and the median RFS was 13 months (Fig. 1A). The 3 year OS rates for patients treated with the original combination (n = 67) versus the modified protocol including lower dose of weekly inotuzumab followed by blinatumomab (n = 43) were 34% (95% CI 23–45%) and 52% (95% CI 36–66%), respectively (Fig. 1B). More patients in Salvage 1 were treated on the modified regimen (blinatumomab addition and lower fractionated inotuzumab; 41/43; 95%) than the initial regimen (38/67; 57%). When outcome was analyzed only in patients who received additional blinatumomab versus no blinatumomab in Salvage 1, with a 2-month landmark (the median time to receiving blinatumomab), the 3 year OS rates were 63% in the modified regimen versus 50% in the original regimen (Additional file 1: Fig. S3; *P* = 0.21). Survival was significantly better among patients treated in Salvage 1 versus Salvage 2+; the 3 year OS rates were 49% (95% CI 37–60%) versus 18% (95% CI 7–33%) (Fig. 1C; *P* = 0.0002). Patients who achieved MRD-negative status at any time also had a better survival. The 3 year OS rate was 54% (95% CI 42–65%) if an MRD-negative status was achieved versus 11% (95% CI 0.8–35%) if it was not (Fig. 1D; *P* = 0.0005).

Overall, 53 of the total 110 patients (48%) had allogeneic SCT in subsequent CR (18 from matched related donors, 17 from matched unrelated donors, 16 from haploidentical donors, 2 with cord stem cells). Twenty-nine of 67 patients (43%) and 24 of 43 patients (56%) underwent allogeneic SCT before and after the amendment of the protocol, respectively (*P* = 0.20). Survival was not improved by performing allogeneic SCT (Fig. 1E). In a 4-month landmark analysis (the median time to allogeneic SCT), the median OS was 47 months for patients who received subsequent allogeneic SCT and 37 months for those who did not. The 3 year OS rates were 54% (95% CI 39–67%) and 54% (95% CI 32–71%), respectively (*P* = 0.98). Among 57 patients who did not undergo allogeneic SCT, 10 (18%) are alive and disease-free after a median follow-up time of 43 months (range, 20–111) (Additional file 1: Table S2). The mortality rates in CR were 26/53 (49%) with SCT and 12/25 (48%) without SCT. The 3 year RFS rates were 51% versus 40% (*P* = 0.67).

Survival by *TP53* mutational status (tested in 60 patients) is shown in Additional file 1: Fig. S4. The 3 year OS rate was 63% (95% CI 46–76%) with wild type *TP53* (n = 41) and 9% (95% CI 0.8–31%) with mutant *TP53* (n = 19) (*P* < 0.0001). Among the 19 patients with *TP53* mutation, 2 are alive, 1 for 9 + months (post SCT) and one for 20 + months (no SCT). Both patients were in the post amendment group and received sequential blinatumomab.

Survival was significantly better among patients with low-risk cytogenetics; the 3 year OS rate was 60% (95% CI 46–71%) in patients with low-risk cytogenetics and 7% (95% CI 0.8–24%) in patients with high-risk cytogenetics (Additional file 1: Fig. S5; *P* < 0.001).

In a post-hoc analysis, we compared mini-Hyper-CVD-inotuzumab ± blinatumomab with our experience with inotuzumab monotherapy (n = 89). The median OS was 17 months with the combination regimen and 6 months with inotuzumab alone; the 3 year OS rates were 40% (95% CI 30–49%) and 11% (95% CI 6–19%), respectively (Fig. 1F; *P* < 0.0001).

Univariate and multivariate analyses analyzed baseline characteristics, treatment modalities, as well as morphologic response and MRD status. Allogeneic SCT was included as a time-dependent variable (Additional file 1: Table S3). By multivariate analysis, the pre-treatment characteristics independently associated with worse survival were: (1) increase peripheral blood blasts percentage [hazard ratio (HR), 1.014; 95% CI 1.006–1.022; *P* = 0.001], (2) high-risk cytogenetics (HR, 2.765; 95% CI 1.606–4.761; *P* < 0.001), and (3) the presence of *TP53* mutation (HR, 2.354; 95% CI 1.318–4.202; *P* = 0.004). In contrast, treatment with lower fractioned doses of inotuzumab followed by blinatumomab was the only independent prognostic factor associated with better survival (HR, 0.574; 95% CI 0.345–0.956; *P* = 0.033).

All 110 patients were evaluable for safety analyses. The treatment was well-tolerated with most side effects being Grade 1–2. Table 3 summarizes all non-hematologic Grade 3–5 adverse events in two or more patients. Early mortality (death within 4 weeks) was noted in seven patients (6%), all of them treated before the amendment of the protocol: four died of infections, one of intracranial hemorrhage, one of SOS, and in one patient the cause of death was unknown. Nine patients died in CR: two died of infections, one of myocardial infarction, one of bronchopulmonary hemorrhage, one of liver graft-versus-host disease, and in four patients the cause of death was unknown. Among the patients who recovered their blood counts, the median time to platelet and neutrophil recovery was 23 and 16 days, respectively, for Course 1, and 22 and 17 days, respectively, for subsequent courses. The median time to platelet recovery was 26 days (range, 12–38 days) before the amendment and 24 days (range, 0–31 days) after the amendment. Overall, 72% of the patients had prolonged thrombocytopenia (platelet count ≤ 50 × 10^9^/L beyond 6 weeks) either during induction (50/110 patients; 45%) or in subsequent courses (48/85; 56%). Fifty seven of 67 patients (85%) and 22 of 43 patients (51%) did not recover their platelet count while on intensification/consolidation in the pre- and post-amendment study groups, respectively (*P* < 0.01). Seventy-three patients (66%) had infections, 15 (14%) had Grade 3–4 increased liver function tests, 25 (23%) had Grade 3–4 hyperglycemia, 12 (11%) had Grade 3–4 increased bilirubin, and 15 (14%) had Grade 3–4 hypokalemia.

**Table 3:** Non-hematologic toxicities

Toxicity	Grade 3, N (%)	Grade 4, N (%)	Grade 5, N (%)
Infections-related and unrelated	49 (45)	17 (15)	7 (6)
Hyperglycemia	21 (19)	4 (4)	0
Pain (back, bone, abdominal, joint, muscle)	19 (17)	0	0
Hemorrhage	14 (13)	1 (1)	2 (2)
Increased liver function tests	13 (12)	2 (2)	0
Hypokalemia	11 (10)	4 (4)	0
Increased bilirubin	10 (9)	2 (2)	0
Cardiac	10 (9)	0	1 (1)
Neurotoxicities	9 (8)	2 (2)	0
Headache	9 (8)	0	0
Fatigue	5 (5)	0	0
Constipation	5 (5)	0	0
Mucositis	5 (5)	0	0
Hepatic sinusoidal obstruction syndrome	2 (2)	0	2 (2) (1 pre SCT, 1 post SCT)
Nausea	3 (3)	1 (1)	0
Hyponatremia	2 (2)	1 (1)	0
Thrombosis	3 (3)	0	0
Generalized muscle weakness	3 (3)	0	0
Hypoalbuminemia	3 (3)	0	0
Hypophosphatemia	3 (3)	0	0
Diarrhea	2 (2)	0	0
Neuropathy	2 (2)	0	0
Pancreatitis	2 (2)	0	0
Acute kidney injury	2 (2)	0	0
Hypercalcemia	2 (2)	0	0
Hypocalcemia	2 (2)	0	0

*SCT* Allogeneic stem cell transplantation

Of the 30 patients who received blinatumomab on study, no patients discontinued blinatumomab due to blinatumomab-related adverse events. One patient (3%) experienced Grade 3 confusion (blinatumomab was held and then dose reduced to 9 mcg/day); one patient (3%) experienced Grade 3 increase of liver function tests, had blinatumomab interrupted, and restarted dose at 9 mcg/day; one patient (3%) experienced Grade 2 cytokine release syndrome. One patient (3%) could not have blinatumomab dose re-escalated to 28 mcg/day due to infusion-related reaction.

SOS occurred in 10 patients (9%) (median age 35 years; range, 19–50 years) after a median of 3 induction/consolidation courses (range, 1–4 courses); six were treated in Salvage 1, two in Salvage 2, and two in Salvage 3 and beyond (Additional file 1: Table S4). SOS was encountered in 7/53 patients (13%) who had subsequent allogeneic SCT versus 3/57 patients (5%) who did not. The median time from the last dose of inotuzumab to the date of allogeneic SCT was 7 weeks on the original study and 14 weeks after the modifications. Three of the seven patients who proceeded to subsequent allogeneic SCT received dual clofarabine and busulfan-based conditioning, 1 received fludarabine-melphalan, 1 received fludarabine-busulfan, and 1 received total body irradiation and etoposide-based conditioning. SOS was noted in 9/67 (13%) on the original study and in 1/43 (2%) on the modified design (*P* = 0.05). Nine of the 10 cases of SOS were fatal, four being directly attributed to SOS, and the six others in the setting of multiple complications post allogeneic SCT.

## Discussion

The combination of mini-Hyper-CVD-inotuzumab ± blinatumomab was effective and safe in patients with R-R ALL. The overall response rate was 83%, and the 3 year OS rate was 40%. In Salvage 1, the overall response rate was 92% and the 3 year OS rate was 49%. These figures emphasize the potential transition of R-R ALL, particularly in Salvage 1, from a disease that carried a death sentence (historical 3 year OS rates < 10%) [1–3] to a disease that carries a reasonable prognosis, particularly if this novel form of therapy is offered to adults with ALL in Salvage 1. These results are unprecedented, emphasizing the need to investigate this regimen in multi-institutional trials. The results compare favorably with historical data of single-agent inotuzumab or blinatumomab in R-R ALL, where the median reported OS were 9 months or less [8, 9].

The longer follow-up of this study in a larger number of patients highlighted two novel important findings. The first is the benefits noted after the addition of sequential blinatumomab and the use of lower and fractionated inotuzumab doses. The 3 year survival rate was 52% (95% CI 36–66%) with the modified regimen versus 34% (95% CI 23–45%) in the original regimen (*P* = 0.16). By lowering and fractionating inotuzumab, by adding ursodiol prophylaxis, and by distancing inotuzumab from allogeneic SCT, the incidence of SOS was reduced from 13 to 2%. Among patients who ultimately underwent allogeneic SCT, the incidence of SOS was reduced from 24% to 4%. Also, the newer strategy still allowed a significant proportion of patients to proceed to allogeneic SCT (56% compared with 43%). The second important finding is the apparent lack of benefit of performing allogeneic SCT, particularly in CR2, and with the modified regimen. This suggests that adding blinatumomab following mini-Hyper-CVD-inotuzumab may offer as much benefit as offering allogeneic SCT after mini-Hyper-CVD-inotuzumab, particularly in Salvage 1. However, despite these maneuvers, the 3 year OS rate was only 49% in Salvage 1. We are currently evaluating the value of measuring MRD by NGS in subsequent CR, and considering CAR T-cell therapy following mini-Hyper-CVD-inotuzumab-blinatumomab in CR. We are also evaluating the incorporation of blinatumomab into the mini-Hyper-CVD-inotuzumab combination (as “dose-dense” therapy rather than sequential blinatumomab). We hope these two modifications might further improve OS in R-R ALL.

In this study, the best results were obtained in Salvage 1 with achievement of MRD-negative status. This indicates the importance of considering such combinations of all effective therapies in the form of a “total chemo-immunotherapy regimen” rather than losing the opportunity of a high “treatment value” and using single-agent antibody therapy, as is currently often practiced in the oncology community and in many referral leukemia centers. Rather than conducting randomized trials to evaluate the individual components incorporated into this regimen, we suggest this mini-Hyper-CVD-inotuzumab-blinatumomab total therapy be investigated in a single arm multi-institutional trial. Once confirmed, the regimen can be modified into a dose-dense regimen with the potential addition of CAR T-cell therapy, if our pilot trials with these 2 modifications are shown to be safe and more effective. In older patients above the age of 70 years, the combination of inotuzumab and blinatumomab is currently being evaluated as an effective chemotherapy-free alternative with a better safety profile.

With the modifications implemented in the latter part of the study (lower and fractionated inotuzumab, ursodiol prophylaxis, distancing the last inotuzumab dose from the allogeneic SCT time, and perhaps more experience), the incidence of SOS was reduced from 13 to 2%, even after allogeneic SCT (SOS 24% vs. later 4%). We suggest incorporating these strategies into future ALL trials evaluating inotuzumab and later allogeneic SCT. The amendments reduced the total inotuzumab dose to a maximum of 2.7 mg/m^2^ (given as fractionated dose, twice per cycles, one week apart). The sequential administration of blinatumomab allowed a deepening of the response (without the risk of relapse which was feared if allogeneic SCT was delayed), as well as increasing the time interval between the last dose of inotuzumab and allogeneic SCT. These favorable SOS results are in contrast with other inotuzumab studies using higher inotuzumab doses, like the INO-VATE trial, and where the administration of more than 2 courses of inotuzumab (> 3–3.5 mg/m^2^) followed by allogeneic SCT was associated with a high SOS rate (20%) and worse outcome [23, 24]. The current strategy also resulted in more patients being able to undergo allogeneic SCT (56% vs. 43%), currently the accepted standard of care, even though it may not be in the future, based on its lack of benefit in our study.

Our analysis identified interesting adverse factors which should be further evaluated in all patients in ongoing trials. These include cytogenetic abnormalities like *KMT2A* rearrangements and low-hypodiploidy/near triploidy, and *TP53* mutations in the R-R setting [25–29]. Low-hypodiploid ALL is highly associated with alterations in *TP53* (91%) [2, 27, 30]. Perhaps combination therapies of low-intensity chemotherapy with inotuzumab and blinatumomab given upfront, followed by CAR T-cell therapy, and the use of other investigational drugs such as anti-CD47 antibodies, could be offered more selectively to patients with R-R ALL and such high-risk features.

In summary, the long-term follow-up of the mini-Hyper-CVD-inotuzumab ± blinatumomab confirmed the efficacy and safety of this regimen and showed that the addition of blinatumomab may further improve outcomes. Confirmation in a large multi-institutional trial is needed in order to establish it as a new form of standard of care therapy in adult R-R ALL.

## Supplementary Information

**Additional file 1: Fig. S1.** Visual schematic of the A original study design and B modified study design. **Fig. S2.** Consort diagram. **Table S1.** Best overall response by salvage status. **Fig. S3.** Overall survival by treatment modality in Salvage 1. **Table S2.** Characteristics and treatments of patients who did not undergo allogeneic stem cell transplantation and remained alive and disease free. **Fig. S4.** Overall survival by TP53 mutation status. **Fig. S5.** Overall survival by cytogenetic risk. **Table S3.** Univariate and multivariate analysis for overall survival. **Table S4.** Characteristics and outcome of patients with hepatic sinusoidal obstruction syndrome.

## Data Availability

The data that support the findings of this study are available from the corresponding author upon reasonable request.
