# Supplementary material for: Results of salvage therapy with mini-hyper-CVD and inotuzumab ozogamicin with or without blinatumomab in pre-B acute lymphoblastic leukemia
Source: J Hematol Oncol. 2023 May 2;16:44. doi: 10.1186/s13045-023-01444-2 (PMC10155451; doi:10.1186/s13045-023-01444-2)

Title: Results of Salvage Therapy with Mini-Hyper-CVD and Inotuzumab Ozogamicin with or without Blinatumomab in Pre-B Acute Lymphoblastic Leukemia

Supplementary Information

Supplemental Tables

Supplemental Table 1. Best overall response by salvage status

Supplemental Table 2. Characteristics and treatments of patients who did not undergo allogeneic stem cell transplantation and remained alive and disease free

Supplemental Table 3. Univariate and multivariate analysis for overall survival

Supplemental Table 4. Characteristics and outcome of patients with hepatic sinusoidal obstruction syndrome

Supplemental Figures

Supplemental Figure 1. Visual schematic of the (A) original study design and (B) modified study design

Supplemental Figure 2. Consort diagram

Supplemental Figure 3. Overall survival by treatment modality in Salvage 1

Supplemental Figure 4. Overall survival by *TP53* mutation status

Supplemental Figure 5. Overall survival by cytogenetic risk

Supplemental Tables

Supplemental Table 1. Best overall response by salvage status

	**S1 (n=79)**	**S1 PR (n=15)**	**S1 CR<12months (n=26)**	**S1 CR>12 months (n=38)**	**S2 (n=17)**	**S3+ (n=14)**
CR	57 (72)	15 (100)	16 (62)	26 (68)	8 (47)	4 (29)
CRp	14 (18)	0	5 (19)	9 (24)	1 (6)	4 (29)
CRi	2 (3)	0	1 (4)	1 (3)	1 (6)	0
Overall	73 (92)	15 (100)	22 (85)	36 (95)	10 (59)	8 (57)
Early Death	1 (2)	0	1 (4)	0	3 (18)	3 (21)
No Response	5 (6)	0	3 (12)	2 (5)	4 (24)	3 (21)

Abbreviations: CR, complete response; CRi, CR with incomplete hematologic recovery; CRp, CR without platelets recovery; S1, salvage 1; PR, primary refractory; S2, salvage 2; S3, salvage 3

Supplemental Table 2. Characteristics and treatments of patients who did not undergo allogeneic stem cell transplantation and remained alive and disease free

Pt	Age (years)	Salvage status	Cytogenetics	Mutations	Time to MRD negativity (days)	Cohort	Treatment	Number of InO cycles	Number of Blina cycles	Status of therapy
1	73	S1	Diploid	NA	74	OD	Mini-HCVD-InO	4	0	Off therapy (subsequent HCVAD and POMP)
2	30	S1	Diploid	NA	18	MD	Mini-HCVD-InO-Blina	2	4	Off therapy (observation)
3	85	S1	Hyperdiploid	NA	136	OD	Mini-HCVD-InO	2	0	Off therapy (observation)
4	29	S3	Diploid	NA	77	OD	Mini-HCVD-InO	3	0	Off therapy (subsequent CAR-T)
5	42	S1	CRLF2::IGH rearrangement	*JAK2*	14	MD	Mini-HCVD-InO-Blina	4	4	Off therapy (observation)
6	71	S1	t(1;6)(q25;q21) and add(7)(q36)	NA	21	OD	Mini-HCVD-InO	4	0	Off therapy (observation)
7	29	S1	Diploid	None	21	MD	Mini-HCVD-InO-Blina	4	4	On maintenance POMP/Blina
8	38	S1	t(1;19)(q23;p13.3)	None	20	MD	Mini-HCVD-InO-Blina	4	4	On maintenance POMP/Blina
9	48	S1	Hyperdiploid	*TP53*, *FLT3* D835Y	32	MD	Mini-HCVD-InO-Blina	4	4	On maintenance POMP/blina
10	57	S1	Insufficient metaphases	NA	65	OD	Mini-HCVD-InO	4	0	Off therapy (subsequent CAR-T)

Abbreviations: Blina, blinatumomab; CAR-T, chimeric antigen receptor T-cell therapy; HCVAD, hyper-CVAD; HCVD; hyper-CVD; InO, inotuzumab ozogamizin; MD, modified study design; MRD, measurable residual disease; NA, not availale; OD, original study design; Pt, patient; S, salvage

Supplemental Table 3. Univariate and multivariate analysis for overall survival

	Univariate			Backward multivariate		
	P	**HR**	**95% CI**	***P***	**HR**	**95% CI**
Age	0.626	1.004	0.989-1.018			
Gender: Female vs. male	0.764	1.076	0.669-1.729			
ECOG PS: 0-1 vs. 2+	0.692	1.130	0.617-2.067			
Salvage: 1 vs. 2+	0.012	1.863	1.147-3.025			
White blood cell count	0.047	1.009	1.000-1.018			
% blasts in PB	0.003	1.011	1.004-1.018	0.001	1.014	1.006-1.022
% blasts in BM	0.059	1.008	1.000-1.017			
CNS leukemia	0.483	1.386	0.557-3.451			
CD19	0.089	0.992	0.983-1.001			
CD20	0.339	0.996	0.988-1.004			
CD22	<0.001	0.979	0.969-0.988			
Cytogenetics risk: Low vs. high	<0.001	3.515	2.137-5.781	<0.001	2.765	1.606-4.761
CRLF2	0.882	1.055	0.523-2.126			
TP53 mutation	<0.001	2.680	1.625-4.419	0.004	2.354	1.318-4.202
Treatment post amendment	0.038	0.588	0.355-0.972	0.033	0.574	0.345-0.956
ASCT (time-dependent)	0.409	0.792	0.455-1.378			

Abbreviations: ASCT, allogeneic stem cell transplantation; BM, bone marrow; CNS, central nervous system; CRLF2, cytokine receptor like factor 2; ECOG, Eastern Cooperative Oncology Group; HR, hazard ratio; PB, peripheral blood

Supplemental Table 4. Characteristics and outcome of patients with hepatic sinusoidal obstruction syndrome

**Parameter**	**Patients**									
Age, (years)	31	19	39	27	30	38	26	46	50	39
Prior ASCT	Yes	No	No	Yes	No	Yes	No	Yes	No	No
Conditioning	Flu/Clo	NA	NA	Flu/Clo	NA	Cy/TBI	NA	Bu/Flu/Clo	NA	NA
Number of cycles	3	2	2	1	4	4	5	3	3	2 + 2Blina
Total InO dose (mg/m^2)	4.4	3.1	3.1	1.8	5.4	5.4	4.3	3.3	3.3	1.5
Blina cohort	No	No	No	No	No	No	No	No	No	Yes
Subsequent ASCT	No	Yes	Yes	No	Yes	Yes	Yes	No	Yes	Yes
Conditioning	CAR-T	Flu/Bu/Clo	Flu/Bu/Clo	NA	Flu/Mel	Flu/Bu	Bu/Clo	NA	Flu/Bu/Cy	Etoposide/TBI
Status	Died	Died	Died	Died	Died	Alive	Died	Died	Died	Died
Causality	Related	NR	NR	Related	NR	NA	NR	NR	Related	Related

Abbreviations: ASCT, allogeneic stem cell transplantation; Blina, blinatumomab; Bu, busulfan; Clo, clofarabine; Cy, cyclophosphamide; Flu, fludarabine; InO, Inotuzumab ozogamicin; NR, not related; TBI, total body irradiation; VOD, veno-occlusive disease

Supplemental Figure 1. Visual schematic of the (A) original study design and (B) modified study design

(A)

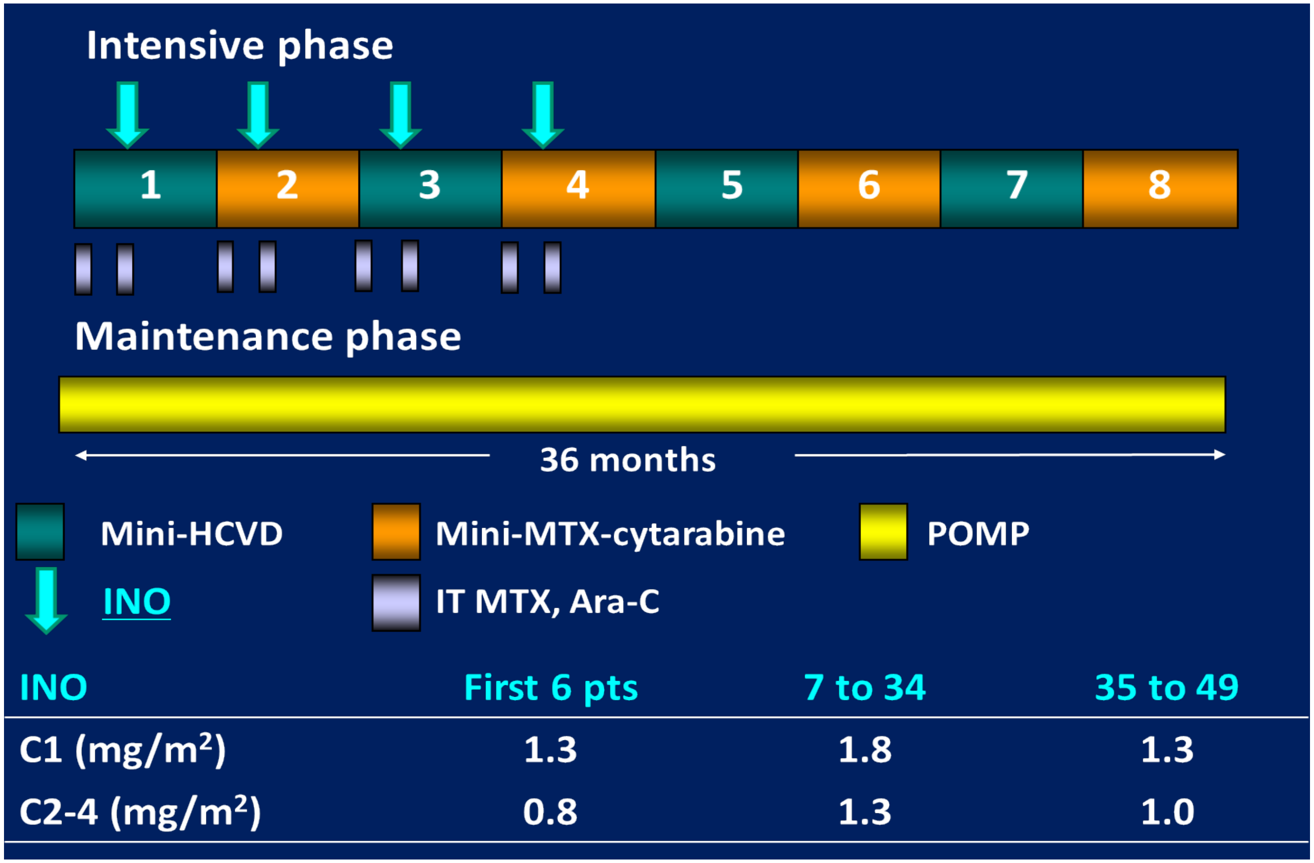

(B)

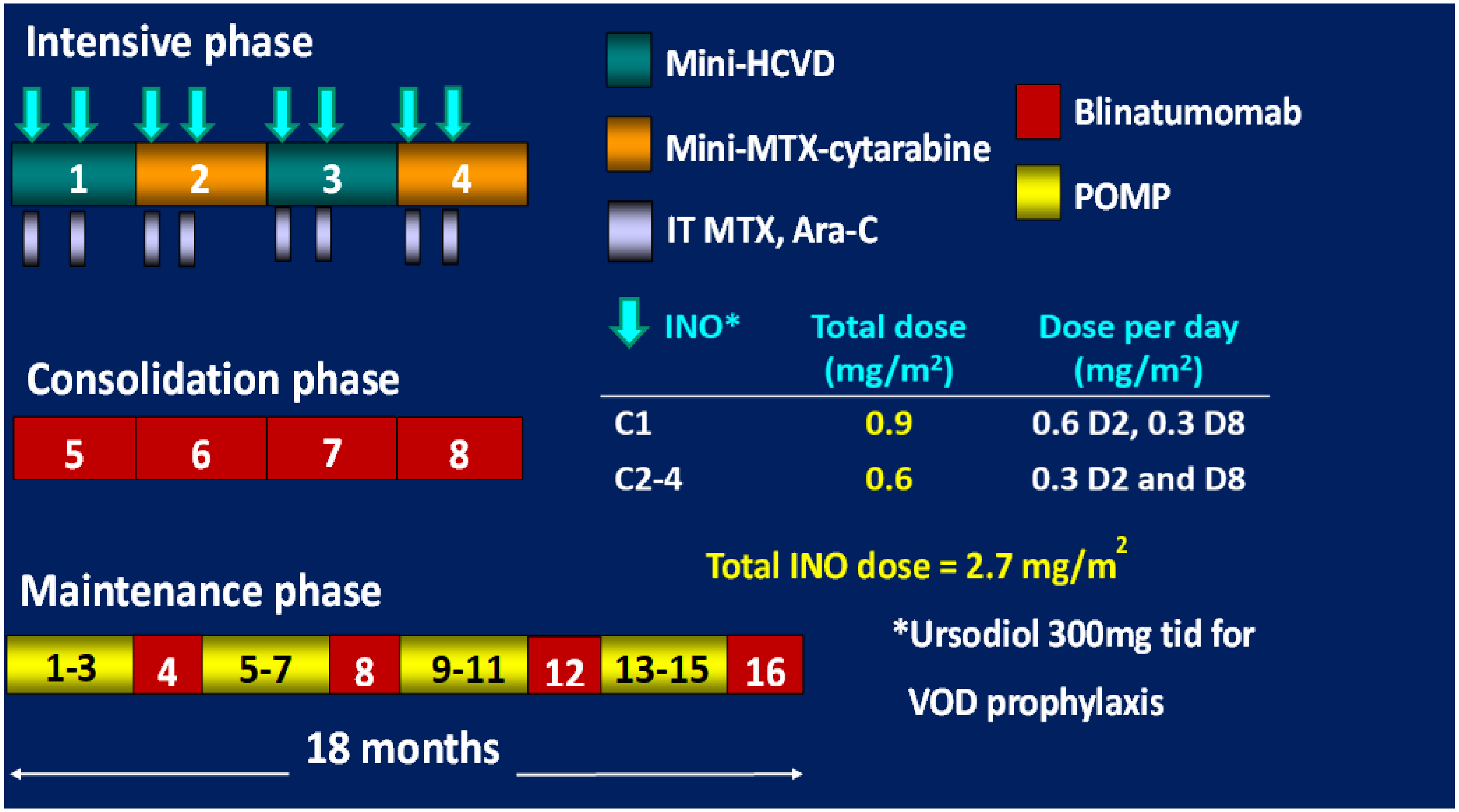
Intensive phase
1
2
3
4
Mini-HCVD
Mini-MTX-cytarabine
IT MTX, Ara-C
Blinatumomab
POMP
INO*
Total dose (mg/m²)
Dose per day (mg/m²)
C1
0.9
0.6 D2, 0.3 D8
C2-4
0.6
0.3 D2 and D8
Consolidation phase
5
6
7
8
Total INO dose = 2.7 mg/m2
Maintenance phase
1-3
4
5-7
8
9-11
12
13-15
16
*Ursodiol 300mg tid for
VOD prophylaxis
18 months

Supplemental Figure 2. Consort diagram

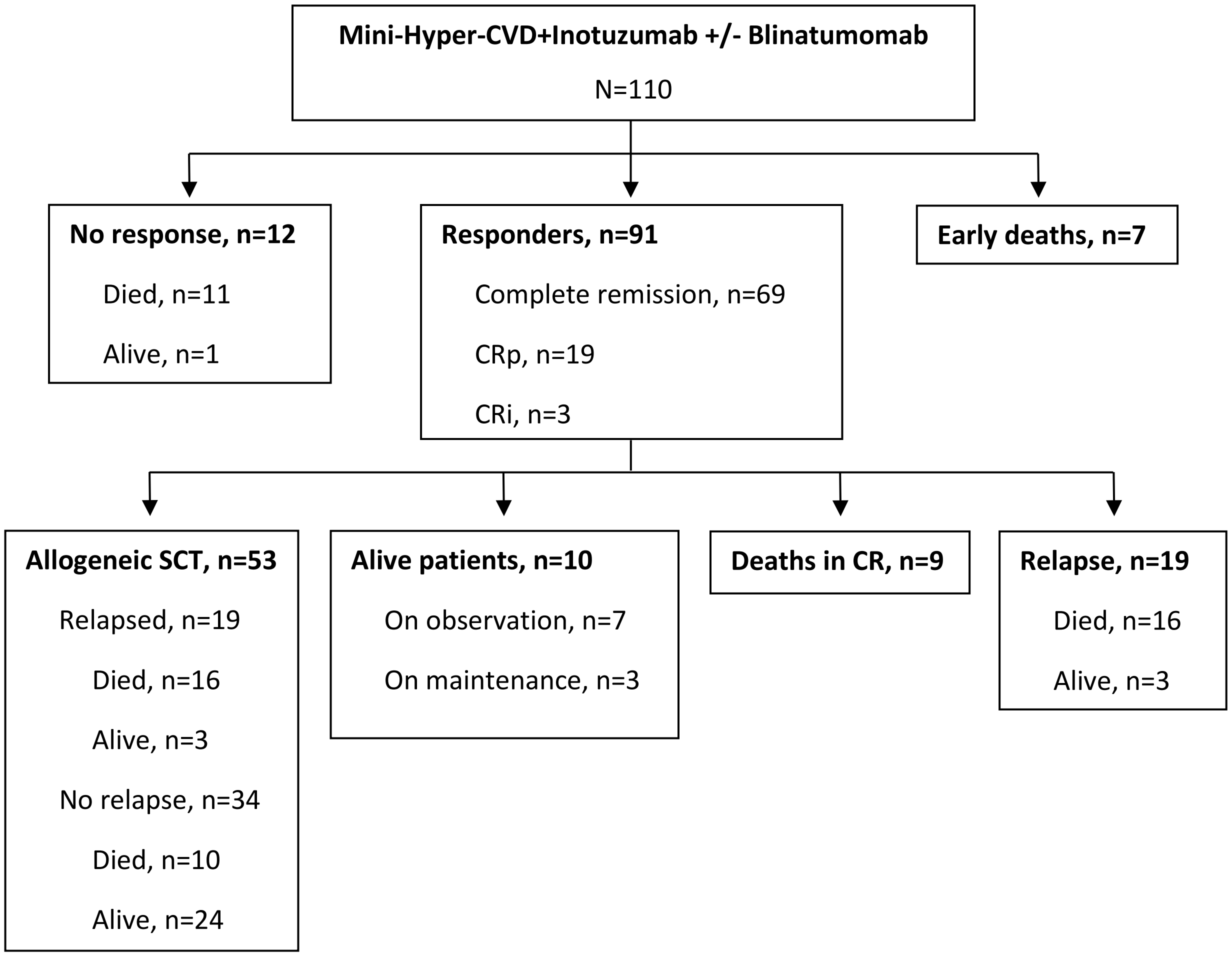

Supplemental Figure 3. Overall survival by treatment modality in Salvage 1

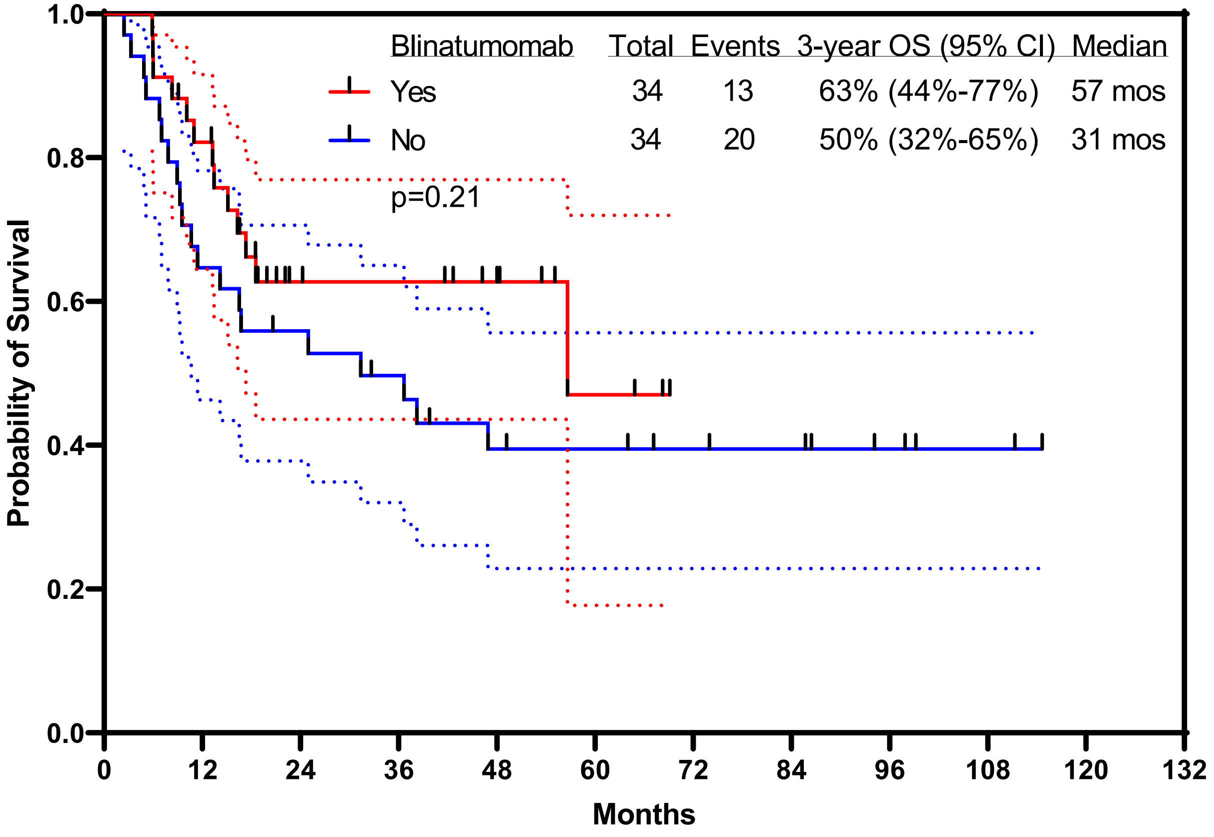

Supplemental Figure 4. Overall survival by *TP53* mutation status

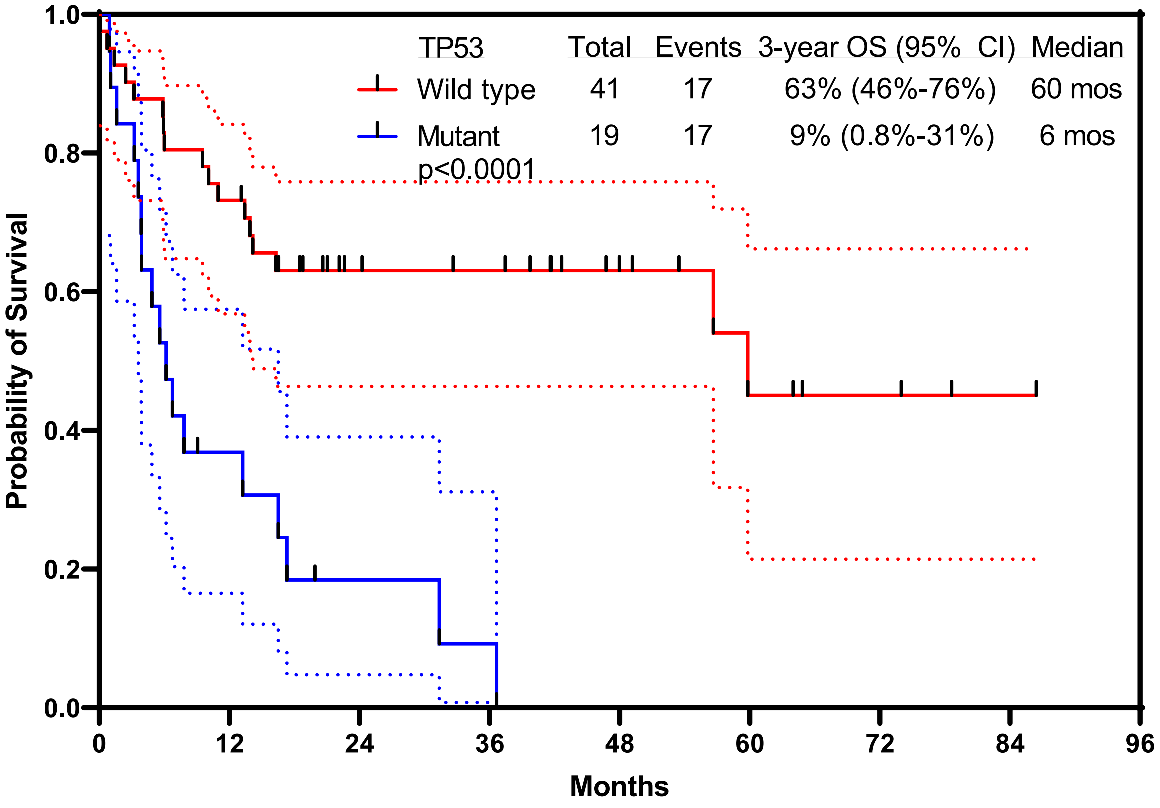

Supplemental Figure 5. Overall survival by cytogenetic risk

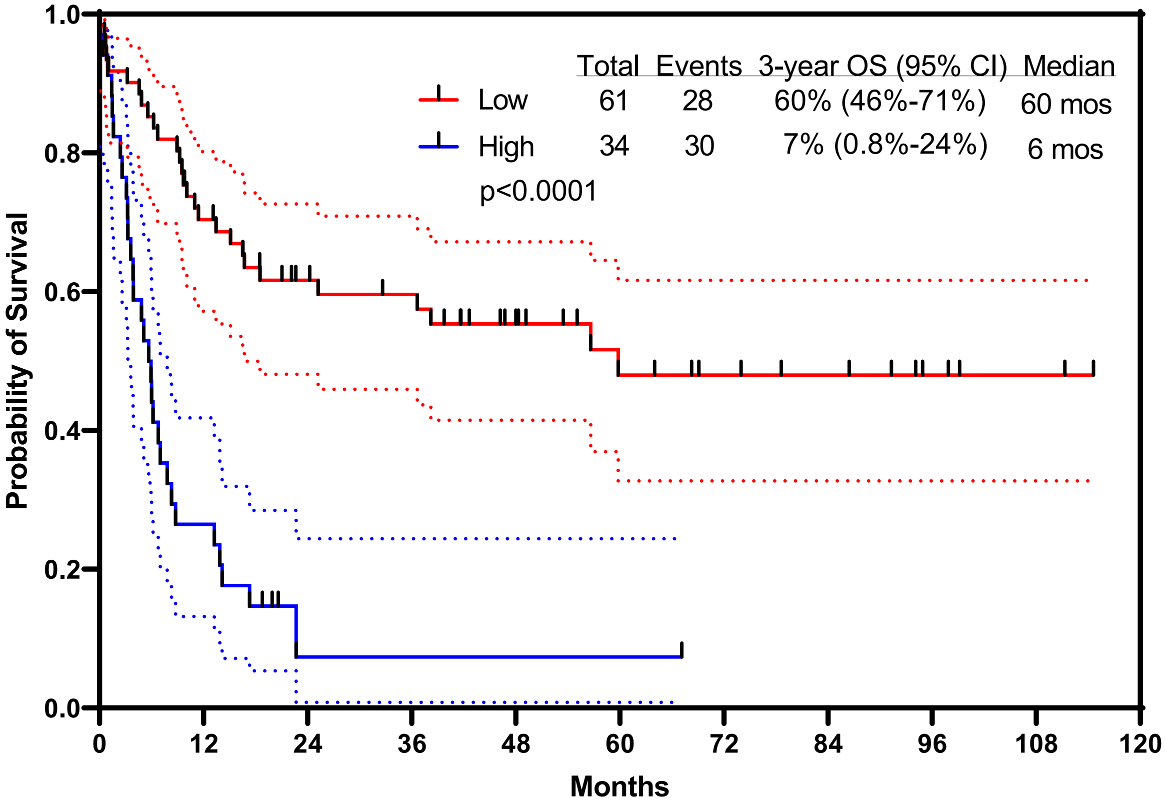

Supplement: Supplementary file 1 — Additional file 1: Fig. S1. Visual schematic of the A original study design and B modified study design. Fig. S2. Consort diagram. Table S1. Best overall response by salvage status. Fig. S3. Overall survival by treatment modality in Salvage 1. Table S2. Characteristics and treatments of patients who did not undergo allogeneic stem cell transplantation and remained alive and disease free. Fig. S4. Overall survival by TP53 mutation status. Fig. S5. Overall survival by cytogenetic risk. Table S3. Univariate and multivariate analysis for overall survival. Table S4. Characteristics and outcome of patients with hepatic sinusoidal obstruction syndrome. [file 13045_2023_1444_MOESM1_ESM.pdf]
